# Verification of automated latex-enhanced particle immunoturbidimetric D-Dimer assays on different analytical platforms and comparability of test results

**DOI:** 10.11613/BM.2020.030705

**Published:** 2020-10-15

**Authors:** Ivana Lapić, Désirée Coen Herak, Snježana Prpić, Andrea Prce, Vanja Raščanec, Renata Zadro, Dunja Rogić

**Affiliations:** 1 Department of Laboratory Diagnostics, University Hospital Center Zagreb, Zagreb, Croatia; 2 Medical Biochemistry Laboratory Dunja Horvat, Primary Care Center Sisak, Sisak, Croatia; 3 Department of Laboratory Diagnostics, University Clinical Hospital Mostar, Mostar, Bosnia and Herzegovina; 4 Medical Biochemistry Laboratory, General Hospital “Dr. Tomislav Bardek”, Koprivnica, Croatia; 5 Medical Biochemistry Laboratory, St Catherine Specialty Hospital, Zagreb, Croatia

**Keywords:** haemostasis, blood coagulation tests, D-Dimer, immunoturbidimetry, validation study

## Abstract

**Introduction:**

The aim of the study was the analytical verification of automated latex-enhanced particle immunoturbidimetric (LPIA) D-Dimer assay INNOVANCE D-dimer on Sysmex CS-5100 and Atellica COAG 360 analysers, and HemosIL D-dimer HS500 on ACL TOP 550, as well as the comparison with the enzyme-linked immunofluorescent assay (ELFA) on the miniVidas analyser.

**Materials and methods:**

Verification included assessment of within-run and between-run precision, bias, measurement uncertainty (MU), verification of the cut-off, method comparison between all assessed assays, and the reference commercial ELFA VIDAS D-Dimer Exclusion II.

**Results:**

Within-run coefficients of variations (CVs) ranged from 1.6% (Atellica COAG 360) to 7.9% (ACL TOP 550), while between-run CVs ranged from 1.7% (Sysmex CS-5100) to 6.9% (Atellica COAG 360). Spearman’s rank correlation coefficients were > 0.99 between LPIAs and ≥ 0.93 when comparing ELFA with LPIA. Passing-Bablok regression analysis yielded constant and proportional difference for comparison of ACL TOP 550 with both Sysmex CS-5100 and Atellica COAG360, and for miniVidas with Atellica COAG360. Small proportional difference was found between miniVidas and both Sysmex CS-5100 and ACL TOP 550. Calculated MUs using D-dimer HS 500 calibrator were 12.6% (Sysmex CS-5100) and 15.6% (Atellica COAG 360), while with INNOVANCE D-dimer calibrator 12.0% (Sysmex CS-5100), 10.0% (Atellica COAG 360) and 28.1% (ACL TOP 550). Excellent agreement of results was obtained, with occasional discrepancies near the cut-off. The cut-off (0.5 mg/L FEU) was confirmed.

**Conclusions:**

The obtained results prove satisfactory analytical performance of LPIAs, their high comparability and almost equal discriminatory characteristics, suggesting them as a valid alternative to ELFA.

## Introduction

D-dimer is a final plasmin-mediated fibrin degradation product consisting of two covalently bound fibrin D domains, cross-linked by factor XIII during clot formation (*1*-*3*).

In conjunction with clinical pre-test probability, measurement of D-dimers is a key non-invasive diagnostic test to rule out venous thromboembolism (VTE) and pulmonary embolism (PE), as well as an aid in the diagnosis of disseminated intravascular coagulopathy (*2*-*5*). However, high D-dimer values are associated with several other conditions related to hyperactivation of blood coagulation and fibrinolysis, such as inflammation, trauma, surgery, pregnancy complications, malignancies, or vascular abnormalities, making their positive predictive value rather poor (*3*, *5*-*7*). In healthy individuals small amounts of fibrinogen are on daily basis physiologically converted to fibrin, resulting in detectable plasma D-dimer concentrations that increase with age (*8*).

Albeit the fact that microplate enzyme-linked immunosorbent assay (ELISA) is still the gold standard method for the quantitative determination of D-dimers due to its highest sensitivity, it is time-consuming, non-automated, characterized by a high level of analytical imprecision, and therefore not suitable for routine practice (*1*). The enzyme-linked immunofluorescent assay (ELFA) is a semi-automated ELISA assay with fluorescence endpoint detection, that exhibits similar sensitivity and specificity to ELISA and is considered the reference commercial D-dimer assay (*1*, *9*). However, the need for rapid turnaround time and the possibility of analysing D-dimers along with other routine coagulation tests on the same analytical platform makes the recently available latex-enhanced particle immunoturbidimetric (LPIA) assays attractive to laboratory professionals (*1*). Numerous available D-dimer assays on the market differ significantly by the specificity of capture monoclonal antibodies used, their reactivity to different fibrin degradation products, method endpoint detection, and calibration standard used, still making standardization of D-dimer assays an unsolved issue (*1*-*3*, *5*, *10*). Thereby, it is of upmost importance to assess the analytical performance of any D-dimer assay prior to implementation into routine practice, as well as the comparability of results, whenever switching from one to another analytical method.

This study was performed with the aim to perform the analytical verification of two automated LPIA D-dimer assays and assess their comparability with the routinely used ELFA method.

## Materials and methods

### Setting and description of assays

The study was conducted at the Department of Laboratory Diagnostics of the University Hospital Centre Zagreb, Croatia.

The INNOVANCE D-dimer assay (Siemens Healthcare, Marburg, Germany) was performed according to the original manufacturer’s application on Sysmex CS-5100 and Atellica COAG 360 coagulation analysers, both produced by Siemens Healthcare, Marburg, Germany. Polystyrene particles covalently coated with 8D3 monoclonal antibody are aggregated in the presence of D-dimer in the sample, which is measured as a decrease of light transmission at 630 nm. For samples with D-dimer concentrations within the measuring range from 0.19 to 4.40 mg/L fibrinogen equivalent units (FEU) results are available within 7 minutes. Automated sample redilution extends the measuring range up to 35.2 mg/L FEU, with a total sample analysis time of 14 minutes (*11*). Since a D-Dimer international standard is not available, INNOVANCE D-dimer calibrator is traceable to a company internal primary master standard containing a pool of patient samples that fulfil the requirements of diagnostic sensitivity and specificity at the cut-off. A secondary master standard is calibrated against it and, following verification of normal range, is used as the product calibrator (*12*). The interference study performed by the manufacturer proved no effect of rheumatoid factor (RF) up to 1330 IU/mL on assay results.

The HemosIL D-Dimer HS 500 (Instrumentation Laboratory, Milan, Italy) is a fully automated LPIA assay that was used on an ACL TOP 550 analyser (Instrumentation Laboratory S.p.A. – Werfen, Milan, Italy). The reagent consists of a suspension of polystyrene latex particles coated with F(ab’)2 fragment of the 8D3 monoclonal antibody that binds the cross-linked D-dimer domain. The degree of agglutination is directly proportional to the concentration of D-dimer in plasma and is measured as the decrease of the light transmitted at 671 nm. Results are available within 5 minutes, if no rerun is performed. The assay measuring range is from 0.22 to 7.65 mg/L FEU, which can be extended by automatic sample dilution up to 128 mg/L FEU, with a final result obtained within 10 minutes from the start of analysis (*13*). The D-dimer HS 500 calibrator is traceable to an internal house standard composed of a pool of patients plasma and assigned according to the proposed harmonisation procedure (*14*, *15*). It is declared that RF does not have an interfering effect on assays results at concentrations below 1400 IU/mL.

The VIDAS D-Dimer Exclusion II assay used on the miniVidas analyser (both from bioMérieux, Marcy-l’Étoile, France) is based on the ELFA principle. It is a two-step enzyme immunoassay that utilizes two monoclonal anti-fibrin degradation products antibodies (10B5E12C9 coated on the solid phase and alkaline phosphatase-labelled antibody 2C5A10) and final fluorescent detection. Contrary to the previous two LPIA assays, analysis of D-dimers by its means is a semi-automated procedure that requires manual sample pipetting in the appropriate test strip well. Results are available within 20 minutes, and the assay is linear from 0.05 to 10.0 mg/L FEU. Additionally, samples with D-dimer concentrations above the upper quantification limit can be manually diluted with the appropriate diluent in the ratio 1:5 and reanalysed, achieving test reporting up to 50.0 mg/L FEU (*16*, *17*).

The recommended cut-off for all assays that were extensively clinically validated for the exclusion of VTE and PE in large cohort studies (*11*, *18*) is 0.5 mg/L FEU. All assays and instruments were used according to their respective manufacturer’s instructions. For each assay, an identical reagent lot was used throughout the study.

### Study protocol

Within-run and between-run precision was determined by analysing assay-specific commercial control samples at a low and high concentration level in triplicate for 5 consecutive days, as proposed by the Clinical and Laboratory Standards Institute (CLSI) EP15-A3 protocol (*19*). INNOVANCE D-Dimer Control 1 and Control 2 (Siemens Healthcare, Marburg, Germany) are lyophilized human plasma based products that contain a determined amount of D-Dimers and were used for the INNOVANCE D-Dimer assay (*11*). HemosIL D-dimer HS 500 Control Level 1 and 2 (Instrumentation Laboratory, Milan, Italy) are ready to use, liquid, human derived control materials (*14*) that were analysed with the HemosIL D-Dimer HS 500 assay.

Bias was calculated from between-run precision data using the following equation: Bias (%) = [(Mean value – Target value)/Target value] x 100.

Measurement uncertainty (MU) was determined by analysing INNOVANCE D-dimer calibrator (lot 561980, target value 4.82 mg/L FEU) on ACL TOP 550 while with both D-dimer HS 500 calibrator (lot B31228, target value 7.34 mg/L FEU) and the proprietary INNOVANCE D-dimer calibrator (lot 561967, target value 4.81 mg/L FEU) on Sysmex CS-5100 and Atellica COAG 360. Measurement uncertainty contributors used for MU calculation included: bias obtained by 10 replicate measurements of respective calibrators, assay repeatability expressed as within-day CV, and calibrator uncertainty declared by the manufacturer, where defined (*i.e.* 4.6% for INNOVANCE D-dimer calibrator). Expanded MU was expressed as a square root of the sum of squares of all MU inputs, multiplied by coverage factor k = 2. Obtained results were compared with the total allowable error (TAE) goal derived from biological variation (*20*). Although MU and TAE concepts differ in their origin, when the MU calculation is based only on a few independent variables, as in our case, the two models become identical and the TAE goal is applicable for evaluation of MU (*21*).

Method comparison was performed as a consecutive study and included parallel analysis of fresh plasma samples with a wide range of concentrations from the daily routine that were analysed on Sysmex CS-5100, Atellica COAG 360, and ACL TOP 550, as well as with the VIDAS D-Dimer Exclusion II assay on the routinely used miniVidas analyser.

The cut-off value of 0.5 mg/L FEU was verified following the protocol defined in the CLSI EP28-A3c document (*22*). A total of 20 apparently healthy volunteers (10 males and 10 females, median age 24 years, ranging from 17 to 46) with no known coagulation defects, recruited from laboratory staff, participated in the study. The cut-off was considered suitable for our patient population if ≥ 90% of results were below 0.5 mg/L FEU.

We also assessed the agreement of D-dimer results obtained with the compared assays, *i.e.* the number of results that were below or above the predefined cut-off value. The agreement of LPIAs with ELFA on miniVidas was assessed for 43 patient samples used in method comparison, while for agreement between LPIAs a total of 63 samples were used (43 from method comparison and 20 healthy controls from verification of the cut-off value). Additionally, results were separately compared for the group of samples with D-Dimer values up to 1 mg/L FEU as well as up to 10 mg/L FEU.

Blood samples were collected into 4.5 ml 0.105 M (3.2%) sodium citrate vacutainers (Becton Dickinson, Plymouth, United Kingdom), centrifuged 15 minutes at 2000xg at ambient temperature, and analysed, whenever possible, within two hours, and no more than four hours from blood collection. All samples used in method comparison were anonymized leftover routine plasma samples otherwise destined for discard. Healthy volunteers that participated in the study gave their informed consent. The study was part of the verification protocol required to be conducted in an accredited laboratory according to the International Standard ISO 15189, and was performed in compliance with the Declaration of Helsinki ethical standards and under the terms of all relevant local legislation.

### Statistical analysis

For precision study performed using commercial control samples, mean values, coefficients of variations (CVs, %), and standard deviations (SDs) were reported. Data normality of patient results used for method comparison was assessed using the Shapiro-Wilk test. Given the non-normal distribution, Spearman’s rank correlation coefficient (ρ) was calculated for the assessment of agreement between compared data. Passing and Bablok regression analysis, assisted by Bland-Altman analysis was used for statistical analysis of method comparisons. The inter-assay agreement relative to the cut-off value was evaluated by the weighted Cohen’s kappa coefficient (κ). Statistical analysis was carried out using MedCalc Statistical Software version 19.1.3 (MedCalc Software Ltd, Ostend, Belgium; https://www.medcalc.org; 2019).

## Results

Results of the precision study and estimation of bias for Sysmex CS-5100, Atellica COAG 360, and ACL TOP 550 analysers are presented in Table 1.

**Table 1:** Precision and bias of INNOVANCE D-Dimer assay on Sysmex CS-5100 and Atellica COAG 360, and HemosIL D-Dimer HS 500 assay on ACL TOP 550

**Assay**	**INNOVANCE D-Dimer**				**HemosIL D-dimer HS 500**	
**Analyser**	**Sysmex CS-5100**		**Atellica COAG 360**		**ACL TOP 550**	
**Control**	**INNOVANCE D-Dimer**		**INNOVANCE D-Dimer**		**HemosIL D-dimer HS 500**	
Level	Control 1 (lot 562246)	Control 2 (lot 562146)	Control 1 (lot 562246)	Control 2 (lot 562146)	Control 1 (lot B31516)	Control 2 (lot B31516)
Target value (mg/L FEU)	0.30	2.69	0.31	2.81	0.60	1.97
Mean ± SD (mg/L FEU)	0.31 ± 0.01	2.77 ± 0.09	0.31 ± 0.01	2.76 ± 0.05	0.62 ± 0.05	2.01 ± 0.04
Within-run CV (%)	3.6	3.2	3.9	1.6	7.9	1.8
Between-run CV (%)	1.7	5.6	6.9	4.3	4.0	1.9
Bias (%)	3.3	3.0	0	- 1.8	3.3	2.0
SD - standard deviation. FEU - fibrinogen equivalent unit. CV - coefficient of variation.						

Obtained MU values for Sysmex CS-5100 and Atellica COAG 360 using D-dimer HS 500 calibrator were 12.6% and 15.6%, respectively, while using INNOVANCE D-dimer calibrator were 12.0% and 10.0%, therefore fulfilling the TAE criteria (*20*) of 28.04%. Measurement uncertainty for ACL TOP 550 using INNOVANCE D-dimer calibrator was 28.1%.

Comparisons of LPIAs on Sysmex CS-5100, Atellica COAG 360 and ACL TOP 550, yielded median values of 1.69 mg/L FEU (interquartile range (IQR): 0.89-6.52), 1.75 mg/L FEU (IQR: 0.91-6.66) and 1.47 mg/L FEU (IQR: 0.93-5.61), respectively. High correlations were demonstrated for all assessed comparisons, *i.e.* Spearman’s ρ for the comparison between Sysmex CS-5100 and Atellica COAG 360 was 0.99 (95% confidence interval (CI): 0.98 to 1.00), for comparison between Sysmex CS-5100 and ACL TOP 550 1.00 (95%CI: 0.99 to 1.00) and 1.00 (95%CI: 0.99 to 1.00) when the results obtained on Atellica COAG 360 and ACL TOP 550 were compared.

**Figure 1:**
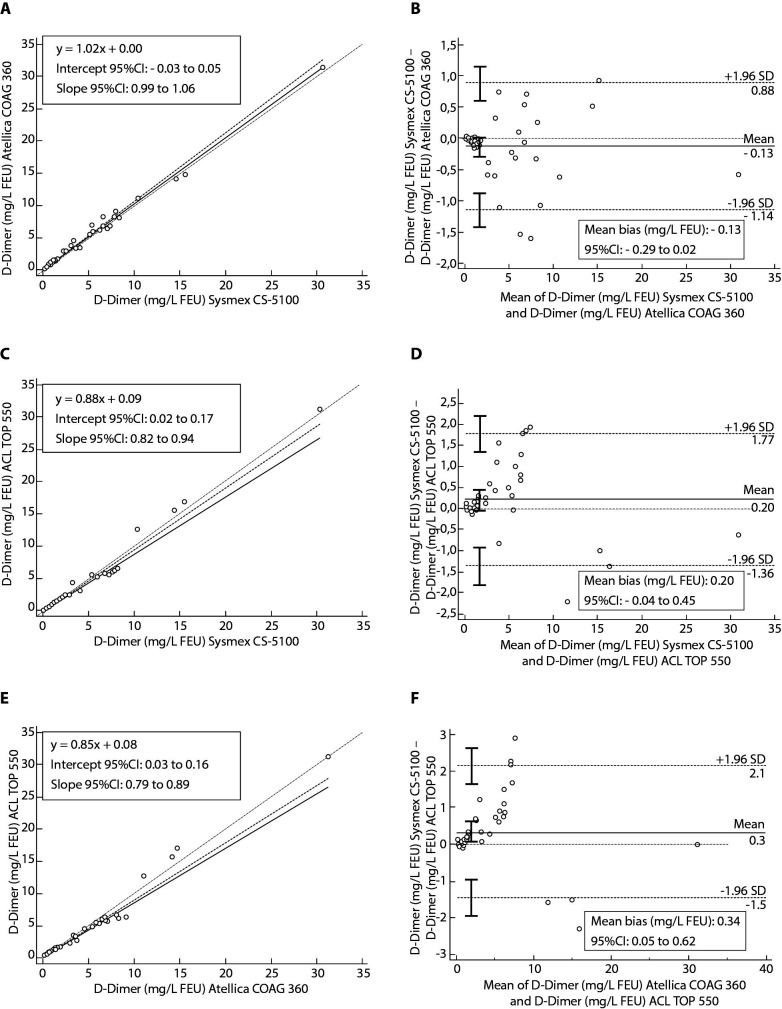
Passing Bablok regression analysis for comparison of D-Dimer results between: (A) Sysmex CS-5100 *vs.* Atellica COAG 360, (C) Sysmex CS-5100 *vs.* ACL TOP 550 and (E) Atellica COAG 360 *vs.* ACL TOP 550. Corresponding scatter diagrams obtained by Bland-Altman analysis with mean biases are shown as B, D and F, respectively. CI - confidence interval. FEU - fibrinogen equivalent unit.

For comparison between Sysmex CS-5100 and Atellica COAG 360, no significant difference was revealed by Passing-Bablok regression analysis, with an equation y = 1.02x + 0 (Figure 1A). Small constant and proportional difference was obtained when results of Sysmex CS-5100 and ACL TOP 550 were compared (y = 0.88x + 0.09) (Figure 1C) and for comparison between Atellica COAG 360 and ACL TOP 550 (y = 0.85x + 0.08) (Figure 1E). The respective mean biases obtained by Bland-Altman analysis were - 0.13 mg/L FEU (95%CI: - 0.29 to 0.02), 0.20 mg/L FEU (95%CI: - 0.04 to 0.45) and 0.34 mg/L FEU (95%CI: 0.05 to 0.62), as shown in Figures 1B, 1D and 1F. By excluding the four samples with D-Dimer values above 10 mg/L the results of method comparison did not differ, yielding similarly high correlation coefficients (the lowest being 0.97 for the comparison between Sysmex CS-5100 and ACL TOP 550), equally negligible differences and mean biases between the evaluated analysers. When considering the 35 samples with values up to 1 mg/L FEU, Spearman’s ρ was 0.97 for comparison of the same D-Dimer INNOVANCE assay between Sysmex CS-5100 and Atellica COAG 360, with no statistically significant differences observed by Passing-Bablok regression analysis with y = 1.04x - 0.01 (intercept 95%CI: - 0.02 to 0; slope 95%CI: 1.00 to 1.09). However, comparisons of Sysmex CS-5100 and Atellica COAG 360 with ACL TOP 550 yielded ρ of 0.868 and 0.881, respectively. Small constant and proportional differences were obtained, with y = 1.24x - 0.12 (intercept 95%CI: - 0.24 to - 0.04; slope 95%CI: 1.12 to 1.52) for Sysmex CS-5100 *vs.* ACL TOP 550, and y = 1.18x - 0.09 (intercept 95%CI: - 0.20 to - 0.02; slope 95%CI: 1.06 to 1.42) for Atellica COAG 360 *vs.* ACL TOP 550. No statistically significant bias using Bland-Altman analysis was observed for either of the latter evaluated comparisons.

Additionally, the results of the method comparison of Sysmex CS-5100, Atellica COAG 360, and ACL TOP 550 with miniVidas are presented in Table 2.

**Table 2:** The results of Passing-Bablok regression analysis, Bland-Altman analysis and Spearman’s rank correlation coefficient for D-Dimer comparison of Sysmex CS-5100, Atellica COAG 360 and ACL TOP 550 with the miniVidas analyser

**Analyser**	**ρ**	**Intercept (95%CI)**	**Slope** **(95%CI)**	**Mean bias, mg/L FEU** **(95%CI)**
Sysmex CS-5100	0.93	- 0.33 (- 0.52 to 0.01)	1.60 (1.27 to 1.82)	- 0.9 (- 1.5 to - 0.3)
Atellica COAG 360	0.95	- 0.27 (- 0.48 to - 0.05)	1.57 (1.34 to 1.85)	1.0 (-1 .6 to - 0.4)
ACL TOP 550	0.94	- 0.16 (- 0.33 to 0.15)	1.37 (1.05 to 1.60)	- 0.7 (- 1.3 to - 0.01)
ρ - Spearman’s rank correlation coefficient. CI - confidence interval. FEU - fibrinogen equivalent unit.				

Moreover, considering the cut-off value of 0.5 mg/L FEU, a 100% agreement of results was observed for comparison between Sysmex CS-5100 and Atellica COAG 360. For other comparisons, minor disagreements were observed, as presented in Table 3.

**Table 3:** Agreement of D-Dimer results relative to the cut-off value of 0.5 mg/L FEU

**Comparison**	**N**	Agreement* (%)	**Weighted Cohen’s kappa coefficient, κ** **(95%CI)**	**D-dimer values of discordant samples** **(mg/L FEU)**		
ACL TOP 550 *vs.* Sysmex CS-5100 / Atellica COAG 360^†^	63	96.8	0.93 (0.84 to 1.00)		ACL TOP 550	Sysmex CS-5100 / Atellica COAG 360
				Sample 1	0.57	0.41 / 0.48
				Sample 2	0.81	0.31 / 0.31
miniVidas *vs.* Sysmex CS-5100 / Atellica COAG 360 / ACL TOP 550^‡^	43	97.7	0.85 (0.55 to 1.00)		miniVidas	Sysmex CS-5100 / Atellica COAG 360 / ACL TOP 550
				Sample 1	0.74	0.33 / 0.31 / 0.38
N - total number of compared samples. CI - confidence interval. FEU - fibrinogen equivalent unit. *Percentage of concordant D-Dimer results between compared analysers relative to the cut-off of 0.5 mg/L FEU. ^†^The same two samples were discordant when comparing ACL TOP 550 with both Sysmex CS-5100 and Atellica COAG 360. ^‡^The same sample was discordant when comparing miniVidas with Sysmex CS-5100, Atellica COAG 360 and ACL TOP 550.						

The two discordant samples, when comparing ACL TOP 550 with Sysmex CS-5100 and Atellica COAG 360, correspond to the healthy volunteers included in the verification of the cut-off, yielding 90% of results below the cut-off. On the contrary, all samples from healthy volunteers analysed on Sysmex CS-5100 and Atellica COAG 360 were below the cut-off. The median D-dimer values were 0.20 (IQR: 0.19 to 0.32) on Sysmex CS-5100, 0.19 (IQR: 0.19 to 0.31) on Atellica COAG 360 and 0.20 (IQR: 0.12 to 0.32) using ACL TOP 550. Consequently, the predefined cut-off was verified for all assays and analysers, and thus can be safely used in routine practice for the patient population served in our laboratory.

## Discussion

This study shows that all evaluated LPIA D-Dimer assays confirm satisfactory precision characteristics and negligible biases that are in compliance with manufacturer’s claimed values, with the exception of within-run precision of ACL TOP 550 for the low concentration level control sample that slightly exceeded the expected imprecision of 6%. This is in concordance with data from earlier validation studies that equally report excellent precision performance of the respective D-dimer assays, with more variable CVs at the low concentration level (*7*, *23*, *24*).

As expected, high between-assay comparability was demonstrated for the same INNOVANCE D-dimer assay applied on two analytical platforms, *i.e.* Sysmex CS-5100 and Atellica COAG 360. High comparability was also evidenced when comparing this assay with the other LPIA HemosIL D-dimer HS500 applied on ACL TOP 550, which can be explained by the fact that both assays utilize the same 8D3 monoclonal antibody. Even the comparison of those immunoturbidimetric assays with ELFA, that is designed along the same principles as the reference microplate ELISA, yielded satisfactory agreement results, *i.e.* correlation coefficients equal or greater than 0.93, with only small constant and/or proportional differences and biases. A larger dispersion of results was observed above the cut-off value and in proportion with increasing D-dimer results. This finding bears no particular clinical significance since D-dimer should not be used for any kind of patient monitoring but for its main indication, which is ruling out VTE or PE. However, in the light of the recent coronavirus disease 2019 outbreak as well as various medical conditions additionally complicated by thromboembolism and inflammation, D-Dimers emerge as a valuable prognostic biomarker, with increasing values being associated with disease severity and higher mortality rates (*25*, *26*). Given the observed differences at higher D-Dimer concentrations, it is a requirement, as for all immunoassays, to provide longitudinal patient monitoring with the same assay whenever possible.

We also obtained clinically acceptable inter-assay agreement between all assessed assays when the recommended cut-off value is used, with occasional discrepancies near the cut-off. Discordance of the one sample with the result above the cut-off with ELFA but below with all LPIAs can be attributed to different methodological principles, *i.e.* ELFA being more specific by using two monoclonal antibodies and a more sensitive detection method, heterogeneity of D-dimers structure, variable reactivity of antibodies to different kinds of fibrin derivatives or cross-reactivity with non-cross-linked fibrinogen and fibrin degradation products (*1*, *9*, *27*). Moreover, observed cases of disagreement regarding the cut-off and occasional different classification of healthy controls with LPIAs imply that different clinical performance can be found even between D-dimer assays based on the same methodological principle. This finding is especially evident when evaluating MU results obtained by analysing the non-proprietary calibrator on ACL TOP 550, indicating that interchangeable use of calibrators might not be possible. However, to get a true insight, it would be valuable to assess the performance of reference material with D-dimer values near the clinical decision threshold.

The present study has some limitations. Firstly, the used TAE goal is probably outdated and not in line with the current state of the art for existing D-Dimer methods and in accordance with improvements in the field. However, more recent data on biological variation in the haemostasis field is still lacking (*1*). Secondly, we are not aware if any of the observed discrepancies between the compared methods is due to analytical interferences, such as RF or heterophilic antibodies (*28*). Finally, a more profound assessment of the cut-off on a population aged over 50 years should be performed to verify the recommended age-adjusted cut-off approach (*29*). Hereby we only verified the applicability of the proposed cut-off concentration of 0.5 mg/L FEU to our population using a small number of qualified reference individuals. This threshold is universally accepted for the exclusion of VTE and PE, based on previous extensive clinical validation studies in large cohorts of patients with suspected VTE and PE where imaging techniques were used for diagnosis confirmation (*11*, *18*).

In conclusion, the results of our study prove the analytical validity of the LPIAs INNOVANCE D-dimer and HemosIL D-dimer HS500, their almost equal discriminatory characteristics, and suggest that they might serve as a valid alternative to the ELFA method. Furthermore, full automation, the possibility of D-dimer analysis on the coagulation analysers simultaneously with other coagulation tests, and shorter turnaround time makes them attractive for use in daily laboratory practice. However, the occasional differences observed once again address the well-known differences between immunoassays, which are for D-dimers additionally complicated by their structure complexity, and highlight the need for further efforts to provide standardization of different D-dimer assays.
